# Influence of Night Shift Work on Heart Rate Variability in an Age- and Gender-Matched Study Group

**DOI:** 10.3390/jcdd11090280

**Published:** 2024-09-07

**Authors:** Kai H. Barckhan, Irina Böckelmann, Stefan Sammito

**Affiliations:** 1 Institute of Pathology, Bundeswehr Central Hospital Koblenz, 56072 Koblenz, Germany; 2 Department of Occupational Medicine, Faculty of Medicine, Otto von Guericke University Magdeburg, 39120 Magdeburg, Germany; 3 Section “Experimental Aerospace Medicine Research”, German Air Force Centre of Aerospace Medicine, 51147 Cologne, Germany

**Keywords:** shift work, autonomous nervous system, heart rate variability, long-term effect, occupational medicine

## Abstract

Regular and long-term shift work is associated with a number of chronic diseases. There is some evidence that shift work also has an influence on the autonomous nervous system. Studies that have examined the effect of shift work on heart rate variability (HRV) have not taken into account age and gender. Therefore, the aim of this study was to investigate the influence of night shift based on 24 h long-term analyses carried out on non-night shift days with a matched control group for age and gender. In total, 172 (128 male, 44 female) healthy shift night workers were compared with subjects from a non-night shift worker group at a ratio of 1:1, forming matched pairs based on the subjects’ sex and age. HRV parameters were analyzed based on 24 h ECG recording. An analysis of the HRV parameters showed only a small difference but without statistical significance between the two groups with regard to all of the HRV parameters examined (SDNN, RMSSD, NN50, pNN50, VLF, LF, HF, LF/HF, DFA1, and DFA2). An analysis of the subgroup of subjects who had only worked night shifts for a minimum of 10 or 20 years, with the respective pairs matched by age and gender, did not reveal any significant differences between the HRV parameters of employees working night shifts and those not working night shifts. Taking into account qualitative aspects of HRV analysis, this study was thus able to show that working night shifts for many years may not have as big an influence on HRV as had been assumed so far.

## 1. Introduction

Regular and long-term shift work is associated with a number of chronic diseases. These include somatic diseases such as type 2 diabetes mellitus, cardiac arrhythmia and coronary heart disease [1,2], hypertension [3,4], and psychological symptoms and diseases [5,6]. Shift work indirectly favors the development of chronic diseases and promotes an unhealthy lifestyle with unbalanced eating habits and physical inactivity [5,6]. In addition, regular shift work contributes to disturbances of the circadian rhythm and related hormone feedback loops and may lead to sleep disorders [6,7,8,9,10,11,12].

The basic rhythm of the cardiac cycle and the control and regulation of the cardiovascular system are determined by efferent fibers of the autonomic nervous system (ANS) via parasympathetic and sympathetic efferent and afferent nerves. A main function of the ANS is to control the cardiovascular system in terms of the adaptive response to external and internal stimuli in order to maintain a constant function of the organism [13]. This also includes the baroceptor reflex sensitivity. External factors can disrupt this balance and, in the long term, lead to disorders of the cardiovascular system and corresponding secondary diseases. Among other things, this results in a change of the barocepter reflex sensitivity and can be analyzed by several heart rate variability (HRV) parameters. Table 1 shows an overview of some of the most used HRV parameters. An analysis of HRV can provide indications of chronic diseases before they manifest [14,15].

Previous studies on the influence of regular night shift work on HRV [16,17,18,19,20,21] have shown that, despite a large dispersion of the results of HRV analysis, there is a trend of a reduction in HRV in connection with night shift work. However, known influencing factors [22,23] have not been sufficiently taken into account in most of the studies on night shift work. Thus, this can lead to a bias in the results, especially if physiological influencing factors that cannot be modified, such as age, sex, and existing (chronic) diseases, are ignored. Furthermore, numerous studies have investigated changes during night shift work [24,25,26,27,28], but conclusions on long-term changes in HRV due to night shift work can only be drawn to a limited extent.

**Table 1:** Overview of the often-used heart rate variability (HRV) parameters, n.o. = no unit assigned [23].

Measure of Variability	Unit of Measurement	Definition and Explanation	Indication	Activity as Part of the Autonomic Nervous System
SDNN	ms	Standard deviation of NN intervals within the measurement area	Short-term and long-term variability	Sympathetic and parasympathetic
RMSSD	ms	Root mean square of successive differences: square root of the arithmetic mean of the squared differences between adjacent NN intervals	Short-term variability	Parasympathetic
NN50	n.o.	The number of pairs of neighboring NN intervals that deviate from one another by more than 50 ms	Spontaneous variability, long-term variability	Parasympathetic
pNN50	%	Percentage of consecutive NN intervals that deviate from one another by more than 50 ms	Spontaneous variability, long-term variability,	Parasympathetic
ULF	ms^2^	Ultralow frequency: power density spectrum below 0.003 Hz	Short-term and long-term variability	No clear assignment
VLF	ms^2^	Very low frequency: power density spectrum in the frequency range of 0.003 to 0.04 Hz	Short-term and long-term variability	Parasympathetic
LF	ms^2^	Low frequency: power density spectrum in the frequency range of 0.04 to 0.15 Hz	Short-term and long-term variability	Sympathetic and parasympathetic
HF	ms^2^	High frequency: power density spectrum in the frequency range of 0.15 to 0.40 Hz	Short-term and long-term variability	Parasympathetic
LF/HF	n.o.	Quotient of the spectrum in LF and the spectrum in HF	Short-term and long-term variability	Ratio or coefficient or ratio between LF and HF band power
DFA1	n.o.	The degree of coincidence/correlation; ranges from 0.5 (coincidental) to 1.5 (correlated) with a normal value of 1.0 and is often used as a non-linear parameter for short NN interval data	Short-term variability	No clear assignment
DFA2	n.o.	Is often used as a non-linear parameter for RR intervals of longer durations of recording, and reduced values are associated with a bad prognosis	Long-term variability	No clear assignment

Therefore, the aim of this paper was to investigate the influence of night shift work on HRV based on 24 h long-term analyses carried out on non-night shift days. We assumed that night shift workers would have a lower HRV on shift-free working days compared to a control group matched by age and sex without night shift work. Furthermore, we hypothesized that this effect would be magnified in the subgroup of night shift workers who have been working night shifts for 10 or 20 years compared to an age- and sex-matched sample.

## 2. Materials and Methods

**Figure 1:**
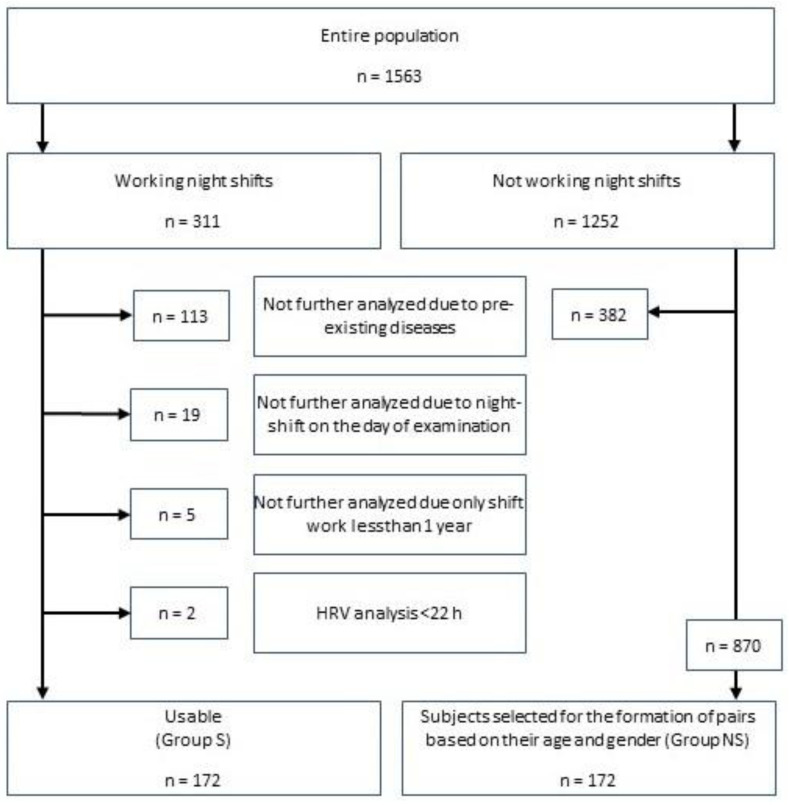
Methodical approach to data selection for the formation of matched pairs.

Based on 24 h HRV analyses in various preventive medicine studies carried out on subjects currently in work and either doing night shift work (*n* = 311) or not doing night shift work (*n* = 1252), male and female study participants aged between 18 and 65 were included in the analysis. Of the 311 subjects who worked shifts with night work (Group S) and had been carrying out this activity for at least one year, 172 could be included in the study (128 male, 44 female). Subjects who suffered from specific pre-existing diseases or to whom circumstances that are known to influence HRV applied were excluded (*n* = 113). The pre-existing diseases leading to the exclusion were identified by means of a questionnaire and included diabetes mellitus, coronary heart disease, history of cardiac infarction, history of coronary artery stent placement, hypertension, other heart diseases, history of cerebrovascular accident, psychiatric medication, thyroid diseases, Parkinson’s disease, epilepsy, chronic obstructive pulmonary disease, chronic renal insufficiency, regular headaches, and pregnancy. Subjects with a HRV analysis less than 22 h (*n* = 2), subjects who had worked night shifts for less than one year (*n* = 5), and subjects who had worked night shifts on the day of examination (*n* = 19) were also excluded. The included participants worked as police officers (*n* = 49), in the rescue system (*n* = 36), as soldiers (*n* = 30), as employees in the communal transport services (*n* = 17), and as other professionals (*n* = 40) and had different forms of night shift duty. After the exclusion criteria had been applied, the subjects of this night shift worker group were compared with subjects from the non-night shift worker group (Group NS) at a ratio of 1:1, forming matched pairs based on the subjects’ sex and age. Subjects with an age difference of ±1.5 years were considered to be of the same age. Care was taken that the subjects of the non-night shift worker group did not have any of the above-mentioned pre-existing diseases either. Figure 1 shows an overview of the methodological approach.

Prior to their participation in the study, all subjects received written and oral information on the planned examination, following which they gave written consent. They were then given the questionnaire for recording possible factors influencing HRV. This was followed by a 24 h ECG measurement, which was taken using a portable ECG recorder (Schiller MT-101, Schiller AG, Baar, Switzerland [*n* = 296]; Medilog Darwin2, Schiller AG, Baar, Switzerland [*n* = 19]; and Tracker I and III recorders, Reynolds Medical, Hortford, UK [*n* = 29]; sampling rate: 1000 Hz). After the data had been recorded, they were analyzed using the original program on a personal computer. Long-term ECGs were analyzed both by a computer and manually by a physician. After the NN intervals had been exported, an HRV analysis was performed with Kubios HRV version 2.0 (University of Kuopio, Kuopio, Finland) [29] with an artifact correction (settings: “custom” and “0.3”). Using the artifact correction option, artifacts due to ectopic beats, missed beat detections, etc. were corrected, which removed the artifacts but did not distort normal RR intervals. Detected artifact beats were replaced using cubic spline interpolation [29,30]. The exact methodological description of the HRV analysis was analogous to the recommendations in valid guidelines [23,31], as well as in accordance with the published procedure for the software [30]. Standard settings for the HRV analysis in the spectral parameters (VLF: 0–0.04 Hz, LF: 0.04–0.15 Hz, and HF: 0.15–0.4 Hz using fast Fourier transformation; window width: 256 s, window overlap: 50%) and for detrended fluctuation analysis (DFA; limits of the short-term (N1) and long-term (N2) fluctuations of N1 = 4–12 and N2 = 13–64 beats) were applied.

The HRV parameters used for the group comparison (SDNN, RMSSD, NN50, pNN50, VLF, LF, HF, LF/HF, DFA1, and DFA2, all analyzed with a fast Fourier transformation) were statistically evaluated using the IBM SPSS 24 software package (IBM, Armonk, NY, USA). Due to the lack of normal distribution based on the Kolmogorov–Smirnov test, descriptive data are presented as median with minimum and maximum values and/or interquartile range (IQR). Differences between the two matched groups were examined by means of the Wilcoxon signed rank test for paired samples with a primary significance level of *p* < 0.05. Due to multiple tests and to avoid alpha accumulation errors, a Bonferroni correction and a corrected significance level of p* < 0.005 (0.05/10 parameters) were applied for the analysis of the HRV parameters. To determine the influence of several variables (such as the number of years and the subject’s group) on the HRV results, the general linear model (GLM) was used for calculations. The effect size was assessed on the basis of the following principle: partial ETA squared (η^2^) < 0.06 corresponds to a small effect, partial η^2^ = 0.06 to 0.14 corresponds to a medium effect, and partial η^2^ > 0.14 corresponds to a large effect.

The individual preventive medicine studies have been approved by the Ethics Committee of the Otto von Guericke University Magdeburg (reference numbers of the approvals of the Ethics Committee: 28/01, 71/08, 132/10, 02/12, 61/13, 63/13, 67/13, 56/14, 24/14, and 65/08). The project is being processed within the scope of the “Magdeburg database about the influence of gender and age on heart rate variability study (MIGA-Heart-Study)” and has been approved separately (139/12).

## 3. Results

The median age was 39.5 years (min.: 20.5 years, max.: 60.9 years, IQR: 17.2 years) for the non-night shift worker group (NS) and 40.2 years (min.: 20.2 years, max.: 60.6 years, IQR: 16.3 years) for the night shift worker group (S). The median difference between the matched pairs was 0.03 years (min: −1.29 years, max.: 1.41 years, IQR: 0.60 years). The median of the underlying recording time of the 24 h ECG measurement taken on a non-night shift day was identical for both groups in the minutes range (NS: 23:59:47, min.: 22:00:00, max.: 23:59:59, IQR: 0:10:23 vs. S: 23:59:06 h, min.: 22:22:32, max.: 23:59:57, IQR: 0:17:03). In Group S, the subjects had worked shifts with night work for a median of 18 years (min.: 1 year, IQR: 14.3 years) up to a maximum of 41 years.

An analysis of the HRV parameters showed a small difference but without statistical significance between the two groups with regard to all of the HRV parameters examined (SDNN, RMSSD, NN50, pNN50, VLF, LF, HF, LF/HF, DFA1, and DFA2; p* > 0.005 for each parameter). The night shift worker group tended to have lower HRV parameters, especially in the time domain parameters. An analysis of the subgroup of subjects who had only worked shifts for a minimum of 10 or 20 years, with the respective pairs matched by age and sex, did not reveal any significant differences between the HRV parameters (see Table 2) of employees working night shifts and those not working night shifts. Also, in this analysis, the night shift worker groups tended to have lower HRV parameters. The variance analysis showed that the years of shift work and the subject’s group only had a small effect (see Table 3).

## 4. Discussion

In summary, the hypotheses put forward could not be confirmed. However, for all ten HRV parameters examined, the data collected showed a trend of lower HRV parameters but without significant differences between employees working night shifts and those not working night shifts. In a sub-analysis of subjects who had been working night shifts for 10 or 20 years, the same results were found. The variance analysis showed only a small effect with regard to the years of shift work and the group (NS or S) as such. Also, a comparison of the HRV parameters of both groups and a sub-analysis with published reference values for HRV parameters [32] indicated that the results were all inside the normal distribution of these parameters.

These results show a certain contradiction to previously published studies, the majority of which observed greater effects of night shift work on HRV [16,17,24,25,26,27,33,34,35,36,37,38,39,40,41,42,43,44,45]. However, there have been some studies [46,47,48,49] that did not show any impact of night shift work on HRV. In this context, a distinction must also be made as to whether the studies investigated direct (very short-term) or indirect (rather long-term) effects of night shift work. For example, some studies have analyzed the intra-individual difference between night shift workdays and days off or non-night shift workdays [16,33,34,35,36,37,46,47,48]. This is mainly aimed at demonstrating the direct effects of night shift work on HRV. The direct influence of night shift work on HRV shown in such studies is thus not surprising. 

Studies that compare individuals who work night shifts and individuals who do not work night shifts but take the measurement on a night shift workday (or the following night) are also mainly aimed at analyzing the direct effects of night shift work on HRV [17,25,26,27,40,42,43]. Here, too, it is to be expected that an influence on HRV would be found. 

To investigate the long-term effects of shift work on HRV, HRV analyses of night shift employees carried out on days off (to avoid including the direct effects of night shift work) must be compared with those of non-night shift employees. However, the design of the few known studies on this subject [24,41,44,45] did not provide for the examination of groups matched according to their age and sex. For example, a study was recently published on the influence of shift work on 203 hospital employees. It found a significant influence of shift work on one HRV parameter compared to the significantly smaller control group of 54 people and a correlation between HRV parameters with mental stress, mood, and sleep status [45]. Although this study was formally matched by sex (because only female subjects were included), the control group was significantly older, so the higher HRV values in the shift worker group could possibly also be due to the younger age. As age and gender are physiological and non-modifiable factors that influence HRV [22,50,51,52,53,54,55,56,57,58,59,60], it is imperative that they are taken into account. From our knowledge, this study is one of the first that has achieved this with a matched study population.

There are particular strengths and some weaknesses of the presented study. This study took physiological factors like age and sex into account in a group of 172 matched subjects. Due to the matched samples, both age and sex could be negated as influencing factors. 

Besides the matching of the two groups, the fact that the recording and analysis methods used for the HRV parameters were the same for all subjects and that measurements were taken over a long period of time (24 h) constitutes a second strength of this paper. This meant that other factors influencing HRV, including the circadian rhythm [61], which would have to be taken into account for short-term measurements, could be excluded. On the other hand, the analysis over the entire 24 h cannot provide any information about a possible impairment of the day/night cycle. Future analyses should therefore also include an analysis of HRV during the day, e.g., from 2:00 pm to 6:00 pm, and during the night, e.g., from 12:00 am to 04:00 am, as has already been carried out in similar ways in other studies [62,63,64], taking into account confounders such as age and sex and with consideration of a personal activity protocol of the participant.

Another aspect that must be emphasized is the wide range of employees from different occupational groups, as this means that the study also included older employees. Including diseases as exclusion criteria for the subsequent analysis is also an advantage, as this meant that their influence on HRV, which in most cases is negative, was not included in the statistical analysis. The fact that data sets from a total of 1563 subjects were available as a population is another advantage, as this made it possible to match the groups of subjects in a very precise manner.

However, it must be taken into account that, naturally, only subjects in work were examined. For this reason, it is to be assumed that the “healthy worker effect” [65] had an influence on the group of subjects the study is based on. This may also have influenced the result of the sub-analyses. Former shift workers who were no longer able to work shifts due to adverse effects on their health may have switched to daytime jobs or may have left working life altogether. However, the size of this effect in the group of subjects examined cannot be quantified.

A further limitation of the present study is that the overall number of test subjects can still be categorized as low. Even though 172 matched subjects were included in the overall analysis, only 93, or 46 matched subjects, could be included in the sub-analyses. This limits the validity of the present study, as significant results may not yet have been recognizable due to the small sample size. Future studies should therefore include as large a sample size as possible in order to avoid this limitation. In fact, as our hypothesis also suggested, a reduced HRV should be found in the group of shift workers, which is also associated with an increased probability of the occurrence of secondary diseases. HRV is particularly suitable as a predictor for cardiovascular diseases long before the diseases manifest [66] and is also reduced in manifested diseases, which is associated with increased mortality and morbidity [23]. By excluding diseases that had already manifested themselves as part of the inclusion and exclusion criteria, an attempt was made to reduce the corresponding bias. This was carried out because, in individual cases, it is not possible to distinguish whether the illness is a consequence of shift work or whether it occurred independently of it. This may also explain the contradictory results in the literature.

In addition, different job requirements may have had an influence on the HRV results because no distinction was made between less stressful and physically or mentally stressful work in the individual groups. It must also be taken into account that only volunteers, who may have a greater basic interest in their health, participated in the study. Another weakness of this analysis is the fact that the subjects did not come from comparable employment relationships and situations (such as civil servants and employees). Thus, different shift models (duration of night shifts, forward vs. backward rotating systems, etc.) could not be taken into account in this analysis. This is a challenge outside very large industrial companies because, in most cases, there will not be a sufficient number of subjects (with similar shift systems). 

## 5. Conclusions

In summary, taking into account qualitative aspects of HRV analysis, this study was thus able to show that working shifts for many years may not have as big an influence on HRV as had been assumed so far. To confirm these findings, it would be desirable that further research be conducted that takes account the physiological influencing factors of age and sex as well as HRV-influencing factors of underlying pre-existing diseases. This also includes a focus on long-term HRV analysis that includes the circadian rhythms to analyze changes in HRV over the daytime on a non-shift workday.

## Figures and Tables

**Table 2:** Comparison of HRV parameters of the night shift worker group (S) with those of the non-night shift worker group (NS), data: median (interquartile range). *n* = 172 pairs; p* = Bonferroni corrected significance level, p* < 0.005 (0.05/10 parameters).

	Independence of the Years of Night Shift Work			Min 10 Years of Night Shift Work			Min 20 Years of Night Shift Work		
HRV Parameter	Night Shift Worker Group (S) *n* = 172	Non-Night Shift Worker Group (NS) *n* = 172	p* Value	Night Shift Worker Group (S) *n* = 93	Non-Night Shift Worker Group (NS) *n* = 93	p* Value	Night Shift Worker Group (S) *n* = 46	Non-Night Shift Worker Group (NS) *n* = 46	p* Value
SDNN [ms]	147.8 (42.6)	154.1 (52.7)	0.680	139.6 (40.4)	150.3 (53.7)	0.452	129.2 (51.7)	142.9 (47.6)	0.391
RMSSD [ms]	36.3 (21.4)	37.6 (23.8)	0.741	30.3 (19.8)	34.0 (23.3)	0.889	28.0 (19.0)	33.1 (20.1)	0.441
NN50	11,518 (14,111)	12,789 (14,598)	0.827	8351 (11,537)	9469 (13,622)	0.874	6322 (9436)	8962 (12,681)	0.488
pNN50 [%]	10.9 (13.2)	12.2 (14.5)	0.489	7.1 (11.0)	9.4 (13.4)	0.559	5.6 (10.2)	9.2 (12.6)	0.307
VLF [ms^2^]	16,967 (41,763)	18,745 (37,290)	0.671	18,344 (44,608)	17,967 (33,183)	0.740	16,237 (34,971)	18,605 (27,265)	0.416
LF [ms^2^]	1196 (1162)	1352 (1366)	0.470	1074 (718)	1174 (1392)	0.544	841 (743)	1047 (1396)	0.207
HF [ms^2^]	399 (550)	457 (709)	0.659	340 (461)	351 (524)	0.666	264 (382)	307 (503)	0.772
LF/HF	2.8 (2.3)	3.0 (2.6)	0.892	3.1 (2.3)	3.6 (2.7)	0.151	3.2 (2.9)	3.5 (2.9)	0.731
DFA1	1.33 (0.17)	1.33 (0.20)	0.680	1.33 (0.16)	1.37 (0.20)	0.287	1.33 (0.21)	1.35 (0.22)	0.623
DFA2	0.98 (0.10)	1.00 (0.09)	0.019	0.99 (0.10)	1.02 (0.11)	0.167	1.02 (0.12)	1.04 (0.12)	0.339

**Table 3:** General linear model comparison of HRV parameters taking into account the years of shift work and the subject’s group (NS or S); significant results are highlighted in bold.

HRV Parameter	Corrected Model			Years of Shift Work			Age			Gender		
	F	Sig.*	Partial η^2^	F	Sig.*	Partial η^2^	F	Sig.*	Partial η^2^	F	Sig.	Partial η^2^
SDNN [ms]	4.239	0.007	0.092	0.004	0.951	0.000	2.449	0.120	0.019	0.193	0.661	0.002
RMSSD [ms]	8.022	**<0.001**	**0.160**	1.245	0.267	0.010	8.959	**0.003**	**0.066**	0.471	0.494	0.004
NN50	10.534	**<0.001**	**0.201**	1.143	0.287	0.009	10.104	**0.002**	**0.074**	1.559	0.214	0.012
pNN50 [%]	7.041	**<0.001**	**0.144**	0.965	0.328	0.008	7.729	0.006	0.058	0.266	0.607	0.002
VLF [ms^2^]	4.394	0.006	0.095	1.974	0.163	0.015	0.148	0.701	0.001	6.761	0.010	0.051
LF [ms^2^]	3.831	0.011	0.084	0.867	0.354	0.007	5.344	0.022	0.041	0.513	0.475	0.004
HF [ms^2^]	7.578	**<0.001**	**0.153**	0.965	0.328	0.008	4.897	0.029	0.037	7.047	0.009	0.053
LF/HF	6.684	**<0.001**	**0.137**	0.986	0.323	0.008	0.345	0.558	0.003	17.854	**<0.001**	**0.124**
DFA1	3.625	0.015	0.079	0.925	0.338	0.007	1.816	0.180	0.014	5.687	0.019	0.043
DFA2	2.527	0.060	0.057	0.290	0.591	0.002	2.845	0.094	0.022	0.020	0.887	0.000

Sig. *: corrected significance level of p* < 0.005; partial ETA squared (η^2^) < 0.06 corresponds to a small effect, partial η^2^ = 0.06 to 0.14 corresponds to a medium effect, and partial η^2^ > 0.14 corresponds to a large effect.

## Data Availability

The data are available on request.
